# A Micro-Costing Study of Screening for Lynch Syndrome-Associated Pathogenic Variants in an Unselected Endometrial Cancer Population: Cheap as NGS Chips?

**DOI:** 10.3389/fonc.2019.00061

**Published:** 2019-02-26

**Authors:** Neil A. J. Ryan, Niall J. Davison, Katherine Payne, Anne Cole, D. Gareth Evans, Emma J. Crosbie

**Affiliations:** ^1^Gynaecological Oncology Research Group, Division of Cancer Sciences, School of Medical Sciences, Faculty of Biology, Medicine and Health, The University of Manchester, Manchester, United Kingdom; ^2^Division of Evolution and Genomic Medicine, School of Medical Sciences, Faculty of Biology, Medicine and Health, The University of Manchester, St Mary's Hospital, Manchester, United Kingdom; ^3^Manchester Centre for Health Economics, Division of Population Health, Health Services Research and Primary Care, The University of Manchester, Manchester, United Kingdom; ^4^Manchester Centre for Genomic Medicine, St Mary's Hospital, Manchester University NHS Foundation Trust, Manchester Academic Health Science Centre, Manchester, United Kingdom; ^5^Division of Cancer Sciences, Faculty of Biology, Medicine and Health, NIHR Manchester Biomedical Research Centre, School of Medical Sciences, The University of Manchester, Manchester, United Kingdom; ^6^Department of Obstetrics and Gynaecology, St Mary's Hospital, Manchester University NHS Foundation Trust, Manchester Academic Health Science Centre, Manchester, United Kingdom

**Keywords:** micro-costing, Lynch syndrome, endometrial cancer, genetic testing, screening

## Abstract

**Background:** Lynch syndrome is the most common inherited cause of endometrial cancer. Identifying individuals affected by Lynch syndrome enables risk-reducing interventions including colorectal surveillance, and cascade testing of relatives.

**Methods:** We conducted a micro-costing study of screening all women with endometrial cancer for Lynch syndrome using one of four diagnostic strategies combining tumor microsatellite instability testing (MSI), immunohistochemistry (IHC), and/or *MLH1* methylation testing, and germline next generation sequencing (NGS). Resource use (consumables, capital equipment, and staff) was identified through direct observation and laboratory protocols. Published sources were used to identify unit costs to calculate a per-patient cost (£; 2017) of each testing strategy, assuming a National Health Service (NHS) perspective.

**Results:** Tumor triage with MSI and reflex *MLH1* methylation testing followed by germline NGS of women with likely Lynch syndrome was the cheapest strategy at £42.01 per case. Tumor triage with IHC and reflex *MLH1* methylation testing of MLH1 protein-deficient cancers followed by NGS of women with likely Lynch syndrome cost £45.68. Tumor triage with MSI followed by NGS of all women found to have tumor microsatellite instability cost £78.95. Immediate germline NGS of all women with endometrial cancer cost £176.24. The cost of NGS was affected by the skills and time needed to interpret results (£44.55/patient).

**Conclusion:** This study identified the cost of reflex screening all women with endometrial cancer for Lynch syndrome, which can be used in a model-based cost-effectiveness analysis to understand the added value of introducing reflex screening into clinical practice.

## Introduction

Lynch syndrome is an inherited predisposition to a constellation of different cancers, of which colorectal and endometrial cancer are the most common (1). Estimates of the prevalence of Lynch syndrome among the general population are as high as 300 per 100,000 (2). Lynch syndrome confers a lifetime risk of endometrial cancer of 30 to 40% (3). Endometrial cancer may be the sentinel event in women with Lynch syndrome, providing an early diagnostic opportunity (4). Those found to have Lynch syndrome are offered risk-reducing interventions including colorectal surveillance to reduce cancer-specific mortality (5). A diagnosis of Lynch syndrome enables cascade testing within families and its identification in those who are yet to develop cancer (6). Identified women may be offered prophylactic surgery to reduce their risk of gynecological cancer (7).

Lynch syndrome arises from germline pathogenic variants within the highly conserved mismatch repair (MMR) system. The molecular characteristics of Lynch syndrome-associated cancers enable tumor-based triage and targeted germline sequencing of the MMR genes, commonly performed by next generation sequencing (NGS). A Lynch syndrome-associated tumor classically shows aberrant expression of associated MMR proteins, MLH1, MSH2, MSH6 and/or PMS2, and microsatellite instability (MSI) (8). Loss of MLH1 expression through somatic methylation of the *MLH1* promotor region is a common sporadic event in endometrial cancer; *MLH1* methylation testing is therefore an effective way of reducing the number of women with MLH1 loss by IHC or whose tumors are MSI-H from expensive germline testing (9).

The National Institute for Health and Care Excellence (NICE) recommends universal screening for Lynch syndrome in people with colorectal cancer (10). Universal screening was identified to be a cost-effective use of healthcare resources because of the number of colorectal cancers prevented in family members who are also found to carry Lynch syndrome (11). Screening women with endometrial cancer provides a further opportunity to save lives from Lynch syndrome-associated cancer (12), however, the costs associated with this screening strategy are not known. The aim of this study was to identify and quantify the resource use and costs associated with different diagnostic strategies relevant to screening for Lynch syndrome in an unselected endometrial cancer population.

## Methods

A micro-costing study was performed to identify the resource use and cost per patient of four diagnostic testing strategies for Lynch syndrome in an unselected endometrial cancer population. The study assumed the perspective of the National Health Service (NHS) in England. The direct costs associated with providing each diagnostic testing strategy were identified. The time horizon for identifying the relevant resources use in this study started with the process of gaining informed consent for any Lynch syndrome testing and ended with generating a report of the final diagnostic test result.

### Diagnostic Testing Strategies

Four Lynch syndrome testing technologies that reflect current and emerging national clinical practice (10) were included in this study: microsatellite instability (MSI) testing; immunohistochemistry (IHC); *MLH1* promoter hypermethylation pyrosequencing (methylation) testing; and next generation sequencing (NGS) (13). The technologies used to produce each diagnostic test were conceptualized into representative clinical pathways using a decision tree to provide a structured approach to represent how each technology would form part of a diagnostic testing strategy for a defined population of women with suspicion of Lynch syndrome. The relevant outcome of testing was defined as either Lynch syndrome diagnosed or not diagnosed. The four diagnostic testing strategies were:

**Strategy 1:** Initial tumor triage with MSI followed by germline NGS testing for pathogenic variants of the MMR genes for all those found to have microsatellite instability (MSI-H).
**Strategy 2:** Initial tumor triage with MSI followed by reflex *MLH1* methylation testing for MSI-H tumors, and germline NGS testing for women where the tumor *MLH1* methylation test shows no hypermethylation.
**Strategy 3:** Initial tumor triage with IHC followed by reflex *MLH1* methylation testing for tumors with MLH1 loss. Germline NGS testing for pathogenic variants of the MMR genes for all women whose tumors show MSH2, MSH6 or PMS2 loss, or MLH1 loss where the *MLH1* methylation test shows no hypermethylation.
**Strategy 4:** No initial tumor triage. All women with endometrial cancer undergo direct germline NGS testing for pathogenic variants of the MMR genes.

**Figure 1:**
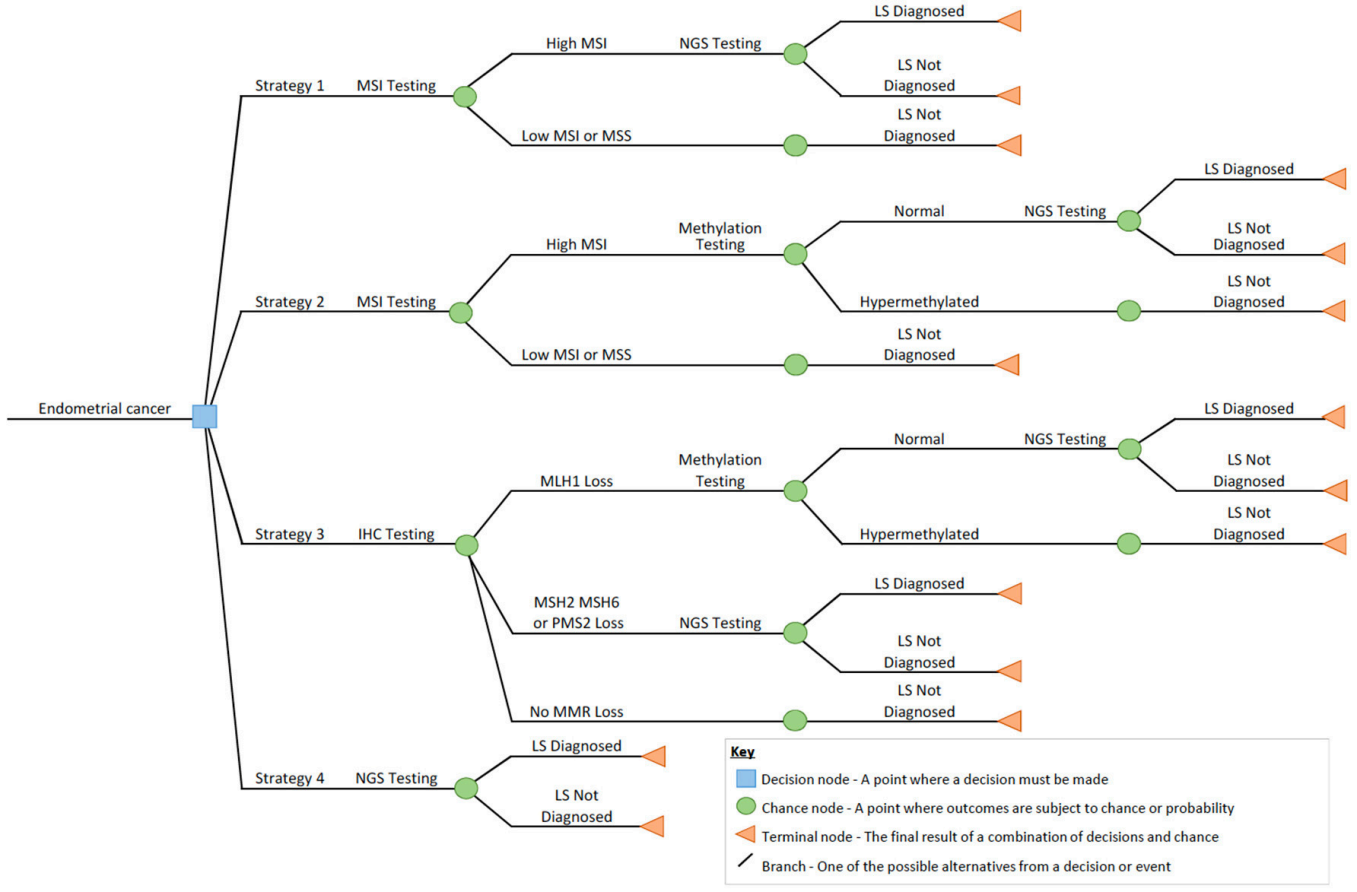
Decision tree outlining the diagnostic strategies for screening endometrial cancer for Lynch syndrome in this study.

The decision tree (Figure 1) was conceptualized through discussion with a panel of 10 local and national experts. These experts included two consultant histopathologists, three senior (>band 8a Agenda For Change pay scale) clinical laboratory scientists, two consultant gynecological oncology surgeons, two consultant clinical geneticists, and one consultant genetic pathologist. The decision tree represented the proportion of cases testing positive with each technology and subsequent impact on the need to conduct further testing. The input values for the decision tree were informed by a pragmatic review of the literature (see Supplementary Appendix 1), which identified the relevant studies to inform the probability of a positive or negative test result in each scenario.

### Identifying Resource Use

The use of resources was identified for each diagnostic testing strategy, assuming the NHS perspective. Resource use included clinical and laboratory staff time, capital equipment, and laboratory consumables.

Clinical and laboratory staff time was collected using a prospective study at a large tertiary genetics and gynecology oncology surgical referral center in the North West of England between 2015 and 2017. The study was approved by the North West Research Ethics Committee (15/NW/0733) and all patients gave written, informed consent to participate. Non-participant direct observation was used to identify the time members of staff dedicated to each element of the diagnostic test process. A single observer recorded timings with a stopwatch over multiple rounds of observation between 2015 and 2017, using a structured data collection tool and recording information on sample batch size and staff pay grade. Participant direct observation was used to record the process of obtaining informed consent for Lynch syndrome testing. Because this was done as part of a research study, all participants were consented prior to any testing. The consent process included discussion about the various tumor tests (MSI, IHC, methylation testing) and indicative germline Lynch syndrome testing.

Hospital information systems were used to derive resource use on capital equipment including diagnostic platforms. Total capital throughput for a given piece of equipment was identified as the total annual number of samples it processed. Total Lynch syndrome-associated capital throughput for a given piece of equipment was identified as the total annual number of Lynch syndrome-associated samples it processed, assuming all newly diagnosed endometrial cancer patients were offered testing.

Consumables were identified from laboratory standard operating procedures and through completion of a structured data collection form by seven laboratory staff who routinely test patients for Lynch syndrome, including biomedical technicians, a consultant clinical scientist, a principal clinical scientist, and clinical genetics technicians.

### Collating Unit Costs

The unit costs (UK sterling; £) for consumables and equipment were extracted from published list prices, or hospital invoices where list prices were unavailable. The unit costs for staff labor were defined as cost per minute using the midpoints of salary grades as per NHS Agenda for Change or the British Medical Association's Hospital Doctor pay scales (see Table S4). The price year for unit costs was standardized at 2017. Table S2 shows the complete list of unit costs and sources.

### Data Analysis

All analysis was carried out using Microsoft Corporation software program Microsoft Office Excel 2011. The base case analysis calculated the direct medical costs of each of the four diagnostic testing strategies shown in Figure 1. The costs were calculated for testing a single sample by multiplying the relevant unit cost (see Table S2) with the relevant items and quantities of resource use.

One-way sensitivity analysis was used to identify the impact of using different estimates for the probability of a positive or negative test result for each diagnostic testing strategy. The ranges of the probability input values were informed by a pragmatic literature search (Supplementary Appendix 1, Table S1) and used to assess the effect different test outcomes would have on the overall cost of each strategy. Two separate scenario analyses explored the impact of the timing of consent on the cost of each diagnostic testing strategy. Two scenarios were explored to understand the cost if consent was taken before any testing, compared with a scenario in which consent was only taken for those needing germline NGS testing.

## Results

Table 1 shows the individual quantities of resource use for MSI, IHC, *MLH1* methylation testing, and NGS, which were used to calculate the total costs incurred per patient for each of the four diagnostic testing strategies.

**Table 1:** Resource use and unit costs.

**Activity**	**Staff member**	**Median staff time per sample (hh:mm:ss)^**◦**^**	**Source of timing (number of samples)**	Staff cost per sample^*^	**Consumable cost per sample (items considered)^**+**^**	**Equipment cost per sample (items considered)^**∧**^**
**ALL**						
Consent for Lynch syndrome testing	BMA: Consultant Yr 3	00:07:39	Direct observation (*n* = 269)	£5.32		
**MICROSATELLITE INSTABILITY**						
Preparation of shavings (>20% tumor)	NHS: 5	00:00:06	Direct observation (*n* = 130)	£0.02		
Preparation of shavings (< 20% tumor)	NHS: 5	00:00:37	Direct observation (*n* = 60)	£0.14		
DNA extraction^#^	NHS: 5	00:02:28	Direct observation (*n* = 16)	£0.54		
DNA extraction^#^	NA	00:00:00	Automated	£0.00		
Clean between sections	NHS: 5	00:01:36	Direct observation (*n* = 13)	£0.35		
Paperwork	NHS: 6	00:03:43	Direct observation (*n* = 13)	£0.97		
Preparation of reagents	NHS: 4	00:01:25	Direct observation (*n* = 48)	£0.26		
0.2ml tubes: label and check	NHS: 4	00:00:42	Direct observation (*n* = 48)	£0.13		
Add reagents and control set up	NHS: 4	00:00:11	Direct observation (*n* = 48)	£0.03		
Spin and place in thermal cycler	NHS: 2	00:00:12	Direct observation (*n* = 48)	£0.03		
PCR cycle 1	NA	00:00:00	Automated	£0.00		
PCR cycle 2	NA	00:00:00	Automated	£0.00		
PCR soak	NA	00:00:00	Direct observation (*n* = 16)	£0.00		
Analysis	NHS: 7	00:01:40	Direct observation (*n* = 39)	£0.52		
Consumables and equipment	–	–	–	–	£19.19 (4)	£0.18 (2)
**IMMUNOHISTOCHEMISTRY**						
Slide myotome MMR	NHS: 5	00:00:44	Direct observation (*n* = 48)	£0.16		
Labeling	NHS: 5	00:01:20	Direct observation (*n* = 48)	£0.29		
Bake	NHS: 5	00:07:30	Direct observation (*n* = 48)	£1.63		
Loading ultra	NHS: 5	00:00:23	Direct observation (*n* = 48)	£0.08		
Automated IHC MLH1	NA	00:00:00	Automated	£0.00		
Automated IHC MSH2	NA	00:00:00	Automated	£0.00		
Automated IHC MSH6	NA	00:00:00	Automated	£0.00		
Automated IHC PMS2	NA	00:00:00	Automated	£0.00		
Unloading ultra	NHS: 5	00:00:21	Direct observation (*n* = 48)	£0.08		
Wash	NHS: 5	00:00:45	Direct observation (*n* = 48)	£0.16		
Dehydration and clear	NHS: 5	00:00:23	Direct observation (*n* = 48)	£0.09		
Slips	NA	00:00:00	Direct observation (*n* = 48)	£0.00		
Checking (1/3 of slides)	NHS: 5	00:00:02	Direct observation (*n* = 48)	£0.01		
Checking blocks	NHS: 5	00:00:06	Direct observation (*n* = 48)	£0.02		
Scoring slides	BMA: Consultant Yr 3	00:00:58	Direct observation (*n* = 48)	£0.68		
Consumables and equipment	–	–	–	–	£12.23 (8)	£0.43 (1)
***MLH1*** **METHYLATION PYROSEQUENCING**						
DNA extraction^#^	NHS: 5	00:02:28	Direct observation (*n* = 16)	£0.54		
DNA extraction^#^	NA	00:00:00	Automated	£0.00		
FFPE bisulphate kit preparation	NHS: 4	00:03:23	Direct observation (*n* = 70)	£0.61		
Lysis of FFPE slice	NHS: 4	00:01:35	Direct observation (*n* = 70)	£0.29		
Bisulphate conversion of DNA	NHS: 4	00:03:06	Direct observation (*n* = 70)	£0.56		
DNA purification and elution	NHS: 4	00:04:05	Direct observation (*n* = 70)	£0.74		
PCR amplification	NHS: 4	00:04:21	Direct observation (*n* = 70)	£0.79		
PCR amplification	NA	00:00:00	Automated	£0.00		
Pyrosequencing	NA	00:00:00	Automated	£0.00		
Data analysis and genotyping	NHS: 6	00:01:34	Direct observation (*n* = 42)	£0.41		
Consumables and equipment	–	–	–	–	£24.25 (11)	£0.23 (3)
**NEXT GENERATION PANEL SEQUENCING OF** ***MLH1, MSH2*****, and** ***MSH6***						
DNA extraction^#^	NHS: 5	00:02:28	Direct observation (*n* = 16)	£0.54		
DNA extraction^#^	NA	00:00:00	Automated	£0.00		
Long range PCR	NHS: 4	00:02:27	Direct observation (*n* = 40)	£0.44		
SequalPrep (Gel transfer)	NHS: 5	00:01:42	Direct observation (*n* = 40)	£0.37		
SequalPrep (Gel)	NHS: 5	00:02:32	Direct observation (*n* = 40)	£0.55		
SequalPrep (Part 1)	NHS: 5	00:01:35	Direct observation (*n* = 40)	£0.34		
SequalPrep (Part 2)	NHS: 5	00:02:08	Direct observation (*n* = 40)	£0.46		
Pooling of patient plates	NHS: 6	00:06:35	Direct observation (*n* = 40)	£1.72		
Library preparation	NHS: 6	00:01:09	Direct observation (*n* = 40)	£0.30		
Thermal-cycling on PCR machine	NA	00:00:00	Automated	£0.00		
PCR clean-up and Qubit check	NHS: 6	00:01:54	Direct observation (*n* = 290)	£0.50		
Library normalization and library pooling	NHS: 6	00:02:12	Direct observation (*n* = 290)	£0.57		
Sequencing	NA	00:00:00	Automated	£0.00		
First analysis	NHS: 5	00:47:00	Direct observation (*n* = 48)	£10.24		
Repeat analysis and reporting	NHS: 7	01:29:00	Direct observation (*n* = 48)	£27.77		
Authorisation of report	NHS: 8A	00:17:00	Direct observation (*n* = 48)	£6.54		
Consumables and equipment	–	–	–	–	£115.14 (45)	£5.43 (2)

* Staff costs taken from NHS and BMA pay scales (see Appendix 3 Supplementary Materials).

+ Consumables and equipment costs taken from laboratory invoices and manufacturer list prices.

∧ Equipment costs per sample consider estimated equipment life span and percentage use for endometrial cancer lynch syndrome diagnostics

# DNA extraction would only needed to be done once per somatic or genomic sample.

◦ *Some activities include periods of automation but only hands-on time is recorded*.

Tumor triage with MSI and reflex *MLH1* methylation testing followed by germline NGS of women with likely Lynch syndrome (Strategy 2) was the cheapest at £42.01 per patient. Tumor triage with IHC and reflex *MLH1* methylation testing of MLH1 protein-deficient cancers followed by NGS of women with likely Lynch syndrome (Strategy 3) cost £45.68. Tumor triage with MSI followed by NGS of all women found to have MSI-H tumors (Strategy 1) cost £78.95. Immediate germline NGS of all women with endometrial cancer (Strategy 4) cost £176.24.

The cost of consenting a woman for Lynch syndrome testing was calculated from 269 directly observed episodes. Two women declined Lynch syndrome testing. On average, the process of gaining consent took 7 min and 39 s (SD: 5 min, 16 s) of consultant time. This cost an average of £5.32.

Immediate, unselected germline NGS testing for pathogenic variants of the MMR genes in all women with endometrial cancer was the most expensive testing strategy. Consumables were an expensive component, costing £115.14 overall, including DNA extraction. As germline DNA is required, it was assumed that all samples for NGS would require *de-novo* DNA extraction from blood. Equipment costs were expensive for NGS, at £5.43 per sample tested. Labor costs were also relatively high due to the complexity of data interpretation, costing £50.35 per sample.

Strategies one to three involved the use of tumor-based triage with IHC and MSI. Tumor based triage by IHC was cheaper than MSI (£21.17 vs. £27.67). The most expensive resource was consumables (£12.23 vs. £19.19 for IHC and MSI, respectively). Labor costs were similar (£8.51 vs. £8.30 for IHC and MSI, respectively); this was despite the need for a consultant grade doctor to interpret the IHC results, because the per-sample time was relatively short. Equipment costs were more expensive for IHC at £0.43 per sample due to use of a dedicated staining platform and associated maintenance costs. These costs were cheaper for MSI testing at £0.18 per sample, using a commercially available kit.

*MLH1* methylation testing was a component of diagnostic strategies two and three, and cost £20.60 and £28.41, respectively, when needed. Methylation testing was cheaper in the context of Strategy two because DNA extraction had already been done for the initial MSI testing. Methylation testing included labor costs at £3.94 (including DNA extraction) or £2.07 (excluding DNA extraction), and equipment costs at £0.23 per sample. Therefore, assuming 30% of endometrial tumors are MSI-H, methylation testing saves £36.95 per patient tested by this strategy because it removes the need for expensive germline NGS by the majority. Incorporating methylation testing in Strategy three (assuming 35% of samples show MMR loss, of which 27% is due to loss of MLH1) reduces the cost of this strategy by £35.21 per patient.

### Sensitivity Analysis

One-way sensitivity analysis explored the potential variation in the cost of each diagnostic testing strategy using pessimistic and optimistic values for the probability of a positive test result and need for subsequent testing (see Supplementary Appendix 2, Table S3). Depending on the source of data, between 22 to 30% of cases in Strategy one require subsequent germline NGS testing (14, 15). The incorporation of *MLH1* methylation testing in Strategy two reduces this proportion to 5–10% of cases (14–16). For Strategy three, MLH1 loss is observed in 16–27% of cases (15, 17), but ~93% of these are due to *MLH1* hypermethylation, meaning that just 7% of those women with MLH1 loss by IHC require germline NGS testing for Lynch syndrome (15). Non-MLH1 protein loss by IHC is seen in 6–8% endometrial tumors and all of these require germline NGS testing (Table S3).

The range of women who were found to carry a pathogenic variant associated with Lynch syndrome in Strategy 2 is between 1 and 3% of cases (14, 15). For Strategy three, 11% of those with MLH1 loss and no *MLH1* hypermethylation tested by NGS will have a pathogenic variant in *MLH1*, according to current literature (15). Twenty percent of those with MSH2, MSH6, or PMS2 loss by IHC will be found to carry pathogenic variants of *MSH2, MSH6*, or *PMS2* (15). Varying the proportions of women requiring subsequent tests in the decision tree, based on pessimistic and optimistic values for the probability of a positive test result in, showed no significant impact on the expected costs for each diagnostic testing strategy.

A scenario analysis explored the impact of the timing of taking consent for Lynch syndrome testing. If consent was only taken at the point of germline NGS testing, the overall cost for Strategy one is cheaper at £75.22 per patient. The overall cost of Strategies two, three, and four would be £36.95, £40.89, and £176.24, respectively. Therefore, taking consent only at the point of germline NGS testing saved the Strategy cost by around £4.52 per person, on average (range £3.73–£5.06).

## Discussion

This study presents the first comprehensive micro-costing analysis of diagnostic strategies for Lynch syndrome testing in endometrial cancer. In total, four pathways were quantified, reflecting the diagnostic strategies relevant to current clinical practice in the UK. The expected costs were £42.01 or £45.68 per case, respectively if MSI or IHC were used for tumor triage and reflex *MLH1* methylation testing was followed by germline NGS of women with likely Lynch syndrome. Immediate germline NGS for all women with endometrial cancer cost £176.24.

Goverde et al. (18) cites estimates close to our calculated costs for three of the diagnostic strategies (MSI €89, IHC €135, *MLH1* hypermethylation €99). However, the cost of NGS was considerably more expensive than our calculation, at €2152 per test (18). Our findings indicate the cost of Lynch syndrome testing in endometrial cancer is sometimes considerably cheaper than previously described. Published cost effectiveness studies to date may have overestimated the cost of Lynch syndrome testing in clinical practice (18–20). In a published model-based cost-effectiveness analysis of screening for Lynch Syndrome in people with colorectal cancer, the estimated unit cost for IHC was £210 and £202 for MSI and £136 per test for *MLH1* hypermethylation. Using NGS for four MMR gene NGS was estimated to cost between £650 and £860 (21). These unit costs were derived from expert estimates from the UK Genetic Testing Network (21). Three model-based cost-effectiveness analysis of unselected endometrial cancer screening for Lynch syndrome base their analysis on these estimates or on insurance charges (18–20). Two of these published studies concluded that using tumor triage with IHC and reflex *MLH1* methylation testing of MLH1 protein-deficient cancers followed by NGS of women with likely Lynch syndrome (our strategy 3) was cost effective for Lynch syndrome screening, despite using higher costs in their modeling(18, 20). One study that used the highest estimated cost for the diagnostic tests indicated that Lynch syndrome screening was not cost-effective (22). These findings are consistent with the observation of Grosse (23) who indicated that the assumed cost of the diagnostic strategy is a key driver of the relative cost-effectiveness of testing for Lynch syndrome (23).

Ours is the first micro-costing study of Lynch syndrome testing in endometrial cancer. Previous micro-costing studies of genetic-based tests have only measured the costs of unselected sequencing of samples (akin to our strategy 4), without quantifying the impact of using tumor based triage (24, 25). Griffith et al (26) micro-costed two gene (*MLH1*/*MSH2*) mutational screening at £1212.17, but this study pre-dates NGS technology and is therefore no longer relevant to clinical practice (26).

Our study was set within a gynecological oncology center comparable with other centers in the UK. It benefited from prospective recruitment and therefore direct observation of 269 patient consent episodes. All but two of 269 patients agreed to Lynch syndrome testing. This is higher than expected from the literature and may reflect testing within a publically-funded healthcare system rather than one based on health insurance, where a positive test result is likely to impact future insurance premiums (27). Taking informed consent is a variable process as each patient encounter is unique; understanding the uncertainty of the process was possible using the multiple observations. We also used direct observations for all non-automated step in the laboratory testing process. The decision tree was conceptualized by a panel of experts with representation from across the diagnostic pathway, therefore reflecting current clinical practice. The sensitivity analysis drew on multiple high-quality studies sourced through an extensive literature search.

To the best of our knowledge, ours may be the first study to micro-cost NGS testing for any indication. Our micro-costing study indicated that analysis of raw NGS output is time consuming and requires considerable expertise. Indeed, 80% of labor costs, and 25% of the overall costs for NGS, were spent on data analysis. It is departmental policy for all NGS results to be analyzed twice, first by an agenda for change (AFC) band 5 and then a band 7 member of staff. Reports are then authorized by a senior member of staff (band 8a). This is for quality assurance purposes and is in keeping with international recommendations (28).

Another key finding was the impact of the timing of patient consent on the overall costs of testing. Consent is fundamental to germline genetic testing since patients have the absolute right to refuse to be tested (29). However, the point at which consent is sought has a considerable impact on the overall cost of Lynch syndrome testing. Consent taken prior to any testing would add this cost uniformly to all strategies. However, if consent were taken only at the point of germline NGS analysis, only a small proportion of women (5–30%) would need to be consented. Somatic tumor analyses are commonplace in histopathology, for example p53 IHC. Consent is not taken for such tests because they are integral to accurate diagnosis, as well as informing prognosis and treatment planning. Crucially such tests make inferences about cancer biology and not the individual's genome. Such an argument could easily be applied for tumor-based Lynch syndrome testing as such tests merely stratify an individual's risk of having Lynch syndrome and do not diagnose a germline condition. Moving the consent to the point of germline testing would require an additional face-to-face meeting with the patient. This could take place in the context of routine cancer follow-up, therefore mitigating the need for an additional appointment and its associated costs. However, any impact of moving consent to the point of germline testing on uptake and health state utility cannot be ascertained from our data, since all patient consents were taken at recruitment into the study, before any testing was carried out.

Our study is limited by the fact that all observations originate from a single site and therefore may not be generalizable. Their application outside England is difficult to assess given geographically distinct populations, diverse health systems and variable costs. We have not accounted for failed tests that need to be repeated at additional cost. It was also not possible to analyse capital costs like heating, lighting, and rent. Capital costs are not insignificant, however the confidentiality implicit in private finance initiatives prevented their incorporation in our analysis. Therefore, the true cost of Lynch syndrome testing is likely to lie somewhere between the cost identified by this micro-costing study, which was as thorough as possible but not exhaustive in its pursuit of all applied costs, and the well-sourced expert estimates, which over-estimate costs to ensure the service does not operate at a net loss (30).

A further limitation of our work is that the proportion of positive test results used in our decision tree originated from populations studied outside the UK and therefore may not be fully representative of the local situation. This is of particular concern given that such patients were tested within insurance-based healthcare systems, potentiating a selection bias in which high risk individuals decline testing to avoid would-be increased premiums. This limitation is unavoidable given the complete lack of UK data relating to the prevalence of Lynch syndrome in endometrial cancer patients.

The extrapolation of our data to inform Lynch syndrome testing in colorectal cancer patients is problematic given their use of BRAF V600E as a proxy of *MLH1* promoter hypermethylation in tumor-based triage (31). Furthermore, it is not clear if the proportions used in our decision tree are transferable to a colorectal cancer population. Nonetheless, the costs of IHC, MSI, and NGS testing (as opposed to the cost of testing strategies) are directly transferable and could be used to improve the robustness of model-based cost effectiveness analysis of Lynch syndrome testing in colorectal cancer.

Regarding tumor-based triage, a strategy using MSI analysis is marginally cheaper than one using IHC as the primary test (£42.01 vs. £45.68). A £3.67 saving per tumor would save the NHS over £33,000 annually, were all 9,000 new diagnoses of endometrial cancer tested for Lynch syndrome. However, the choice of tumor triage is complex and beyond a simple cost comparison; this is especially the case when the cost difference is marginal. Both methods have equitable sensitivity and specificity according to the literature (11). IHC has the advantage of identifying the likely mutated gene, which can aid the subsequent interpretation of sequencing data (32). IHC can be performed in most histopathology departments, whereas MSI requires specialist laboratories. IHC is thought to be more sensitive in the case of those carrying a *MSH6* pathogenic variant, where tumors may not be MSI-H (33). However, interpretation of MMR expression patterns requires consultant pathology expertise, and only MSI can identify missense pathogenic variants in MMR genes whereby the protein is expressed but is not functional (34). The choice of tumor triage depends on the availability of local services, expertise, and infrastructure; these data do not infer that any one strategy is clinically superior to another for the diagnosis of Lynch syndrome.

The importance of our work is its ability to inform healthcare policy. There is a growing call for screening all endometrial cancer patients for Lynch syndrome (12, 35). A potential barrier to the transition from expert opinion to clinical application is cost. To date there have been no micro-costing data available to inform policy makers as to the actual costs of Lynch syndrome testing in the UK. As outlined above, the current estimated costs might be prohibitively high and thus impede the implementation of Lynch syndrome testing in endometrial cancer. Our data should prompt healthcare providers to look at this again.

## Conclusion

We present a micro-costing study for Lynch syndrome testing in unselected endometrial cancer patients from a large tertiary referral center in the North West of England. The use of tumor triage with MSI and reflex *MLH1* methylation testing is the cheapest strategy at £42.01 per case. Substituting MSI for IHC as the initial tumor-based triage increases costs marginally to £45.68. Moving the point of consent for Lynch syndrome testing to just before germline testing reduces the costs of those strategies that incorporate tumor triage. NGS panel testing is considerably cheaper than current estimates at £176.24 per test. The next phase is to use these estimates in model-based cost-effectiveness analysis to understand the relative value of a national Lynch syndrome-testing programme for endometrial cancer.

## Author Contributions

NR recruited to the study and collected data. NR and ND analyzed data and wrote the first draft of the manuscript. AC collected data. EC and KP designed the study, supervised study execution and contributed to data interpretation. DE provided study oversight. EC was Chief Investigator and study guarantor. All authors provided critical comment, edited the manuscript, and approved its final version.

### Conflict of Interest Statement

The authors declare that the research was conducted in the absence of any commercial or financial relationships that could be construed as a potential conflict of interest.

## Abbreviations

NGS: Next generation sequencing
MSI: Microsatellite instability
MSI-H: Microsatellite instability high
IHC: Immunohistochemistry
MMR: Mismatch repair
NICE: National Institute for Health and Care Excellence
NHS, National Health Service: England.
