# Supplementary material for: A Micro-Costing Study of Screening for Lynch Syndrome-Associated Pathogenic Variants in an Unselected Endometrial Cancer Population: Cheap as NGS Chips?
Source: Front Oncol. 2019 Feb 26;9:61. doi: 10.3389/fonc.2019.00061 (PMC6399107; doi:10.3389/fonc.2019.00061)
Supplement: Supplementary file 1 [file Table_1.DOCX]

**A micro-costing study of screening for Lynch syndrome-associated pathogenic variants in an unselected endometrial cancer population: cheap as NGS chips?**

Supplementary Material

Table of Contents

Appendix 1 – Methods supplementary 2

Pragmatic Literature review 2

Table S1 PICO Framework defining inclusion criteria of studies used to inform sensitivity analysis 2

Figure S1- A diagrammatic representation of the timing of consent within the time horizon 4

Table S2 – Costs and sources for consumables 5

Appendix 2- Results supplementary 10

Pragmatic literature review 10

Table S3: Sensitivity analysis inputs from clinical studies identified through a pragmatic literature search. 11

Figure S2: Prism chart of study identification and selection 16

Appendix 3 – Salary source data 17

Table S4: BMA pay scales used to calculate medical doctors’ salary costs. 17

References………………………………………………………………………………………………… 18

# Appendix 1 – Methods supplementary

## Pragmatic Literature review

The aim of this rapid review was to identify the relevant studies to inform the probability of a positive or negative test result in each diagnostic strategy shown in Figure 1.

Method

The rapid review used an electronic search strategy devised and performed by a specialist librarian. MEDLINE, EMBASE, Cochrane CENTRAL and Web of Science were searched in January 2018 using the Medical Subject Headings (MeSH) terms “Colorectal Neoplasms, Hereditary Nonpolyposis” and “Endometrial cancer”.

The PICO framework was used to define the study inclusion criteria (see Table S1). In addition, only studies published in the English language were included. Furthermore, studies had to contain sufficient detail as to devise the outcomes of their diagnostic methodologies. Studies were excluded if data were found to be insufficient, unclear or contradictory. Authors were not contacted for further information. Two independent observers screened titles and abstracts to identify studies for full text review. Study bias was defined with the use of the QUADAS-2 instrument (<http://www.bristol.ac.uk/population-health-sciences/projects/quadas/quadas-2/>)

### Table S1 PICO Framework defining inclusion criteria of studies used to inform sensitivity analysis

| Population | Women with newly diagnosed endometrial cancer |
| --- | --- |
| Intervention | Diagnostic strategies included were tumour triage (through either the use of immunohistochemistry (IHC) for the four MMR proteins or MSI with or without methylation testing) and/or direct germline sequencing |
| Comparator | No testing of endometrial cancer for Lynch syndrome |
| Outcome | The cost of testing endometrial cancer for Lynch syndrome via the included diagnostic strategies of tumour triage (through either the use of immunohistochemistry (IHC) for the four MMR proteins or MSI with or without methylation testing) and/or direct germline sequencing |
| Study type | Aimed to diagnose Lynch syndrome in a secondary care setting |

### Figure S1- A diagrammatic representation of the timing of consent within the time horizon


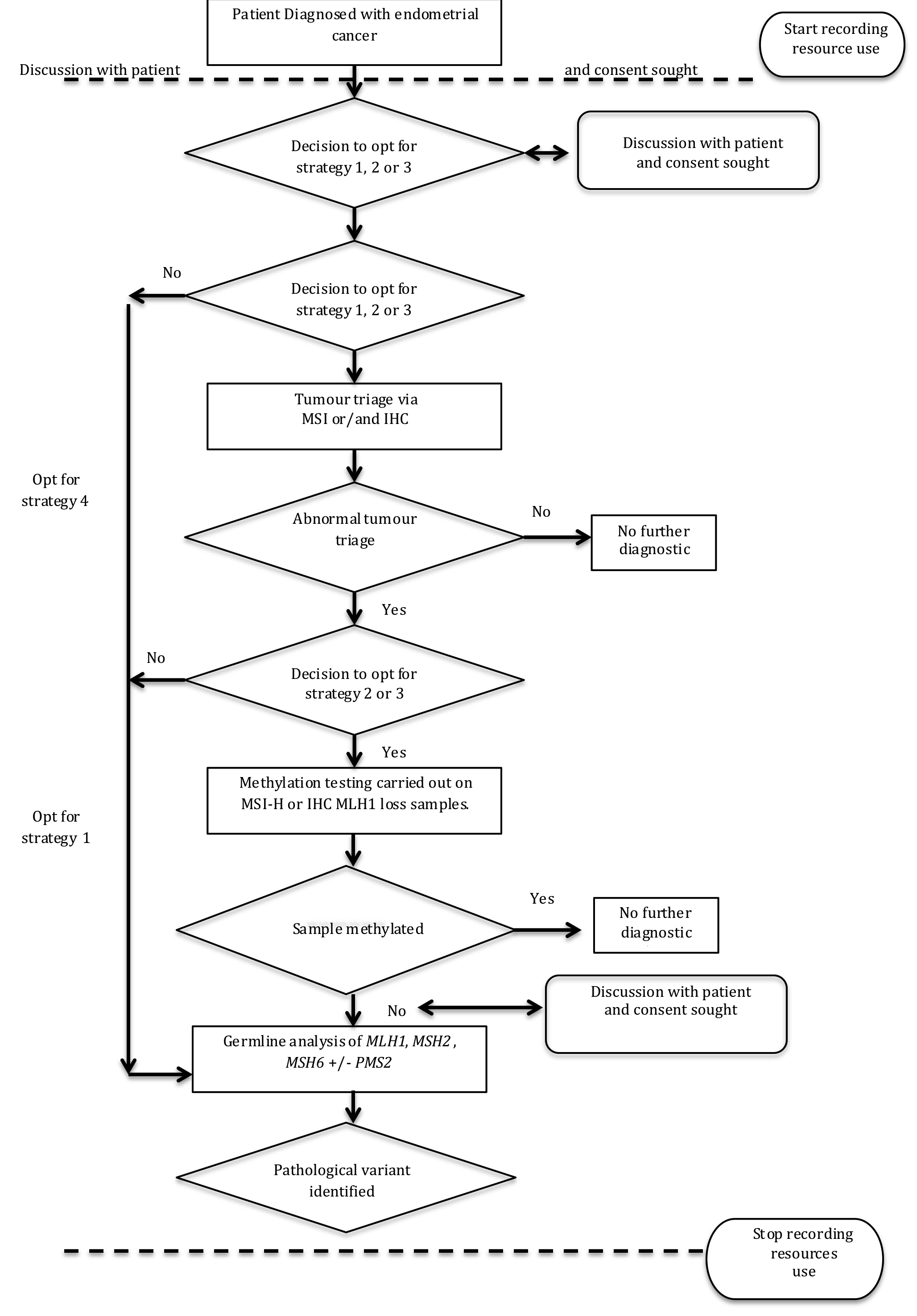


### Table S2 – Costs and sources for consumables

| MSI - Consumables |  |  |  |  |
| --- | --- | --- | --- | --- |
|  |  |  |  |  |
| Item | Manufacturer | List Price (£) | Unit Cost (Per Sample) | Source of Cost |
| EZ1 DSP DNA Blood Kit | Qiagen | £310 | £6.46 | [https://www.qiagen.com/gb/shop/](https://www.qiagen.com/gb/shop/automated-solutions/sample-preparation/ez1-dsp-dna-blood-kit-row/#orderinginformation) |
| QIAamp DSP DNA FFPE Tissue Kit | Qiagen | £230 | £4.60 | [https://www.qiagen.com/gb/shop/](https://www.qiagen.com/gb/shop/automated-solutions/sample-preparation/ez1-dsp-dna-blood-kit-row/#orderinginformation) |
| Scalpel | Swann Morton | £0.33 | £0.33 | Internal invoice |
| MSI Kit | Promega | £780 | £7.80 | [https://www.promega.co.uk/products/](https://www.promega.co.uk/products/molecular-diagnostics/amplification/microsatellite-instability-msi-analysis/?catNum=MD1641) |
|  |  |  |  |  |
| IHC - Consumables |  |  |  |  |
|  |  |  |  |  |
| Item | Manufacturer | List Price (£) | Unit Cost (Per Sample) | Source of Cost |
| MLH1 Abx | Ventana | £225 | £4.50 | Internal invoice |
| MSH2 Abx | Cell Mark | £485 | £1.46 | Internal invoice |
| MSH6 Abx | Cell Mark | £800 | £2.40 | Internal invoice |
| PMS2 Ab | Cell Mark | £184 | £3.68 | Internal invoice |
| Reaction Buffer | Ventana | £82 | £0.00 | Internal invoice |
| Glass slides | Thermo Scientific | £0.12 | £0.12 | Internal invoice |
| Acrytol | Leica Biosystems | £0.02 | £0.02 | Internal invoice |
| Cover slips | Celestron | £0.04 | £0.04 | Internal invoice |
|  |  |  |  |  |
| Methylation - Consumables |  |  |  |  |
|  |  |  |  |  |
| Item | Manufacturer | List Price (£) | Unit Cost (Per Sample) | Source of Cost |
| FFPE Kit | Qiagen | £288 | £5.76 | <https://www.qiagen.com/gb> |
| Disposable Scalpel | Scientific Lab Supplies | £5.72 | £0.57 | Internal invoice |
| Pyromark Gold Q96 reagents | Qiagen | £341 | £2.15 | https://www.qiagen.com/gb |
| Pyromark Q96 plate | Qiagen | £131 | £0.11 | https://www.qiagen.com/gb |
| Pyromark Q96 cartridge | Qiagen | £255 | £4.25 | https://www.qiagen.com/gb |
| Pyromark Annealing Buffer (250ml) | Qiagen | £33.80 | £0.05 | https://www.qiagen.com/gb |
| Pyromark Binding Buffer (200ml) | Qiagen | £31.20 | £0.08 | https://www.qiagen.com/gb |
| Pyromark Washing Buffer (200ml) | Qiagen | £31.20 | £0.23 | https://www.qiagen.com/gb |
| Pyromark Vacuum Prep Filter Probes (100) | Qiagen | £184 | £11.04 | https://www.qiagen.com/gb |
| NaOH (10%) | Fluka | £6.25 | £0.00 | Internal invoice |
| EtOH | Fluka | £111.20 | £0.00 | Internal invoice |
|  |  |  |  |  |
| NGS - Consumables |  |  |  |  |
|  |  |  |  |  |
| Item | Manufacturer | List Price (£) | Unit Cost (Per Sample) | Source of Cost |
| EZ1 DSP DNA Blood Kit | Qiagen | £310 | £6.46 | [https://www.qiagen.com/gb](https://www.qiagen.com/gb/shop/automated-solutions/sample-preparation/ez1-dsp-dna-blood-kit-row/#orderinginformation) |
| MyFi PCR mix (500 * 50ul) | Bioline | £580 | £5.10 | [https://www.bioline.com/](https://www.bioline.com/fr/myfi-mix.html) |
| Biomek AP96 P250 Tips | Beckman Coulter | £61 | £6.11 | Personal contact via email with Beckman Coulter |
| Biomek AP96 P50 Tips | Beckman Coulter | £66 | £6.61 | Personal contact via email with Beckman Coulter |
| Biomek AP96 P20 Tips | Beckman Coulter | £61 | £6.11 | Personal contact via email with Beckman Coulter |
| 1250ul Racked Tips | Sarstedt LTD | £57 | £0.07 | Personal contact via email with Sarstedt |
| 1000ul Filter Tips | Sarstedt LTD | £80 | £0.08 | Personal contact via email with Sarstedt |
| 200ul Filter Tips | Sarstedt LTD | £153 | £0.08 | Personal contact via email with Sarstedt |
| 20ul Filter Tips | Sarstedt LTD | £153 | £0.08 | Personal contact via email with Sarstedt |
| 20ul Filter Tips - Neutral | Sarstedt LTD | £153 | £0.08 | Personal contact via email with Sarstedt |
| Stock DNA Tubes | Fluid X | £485 | £0.51 | Personal contact via email with Fluid X |
| Stock DNA Racks | Fluid X | £102 | £10.20 | Personal contact via email with Fluid X |
| 1000ul Space Saver Tips | Anachem | £34 | £0.04 | [https://www.anachem.co.uk/](https://www.anachem.co.uk/Pipette-Tips/BioClean-Universal-Tips/SpaceSaver/Tips-1000-%C2%B5L-768-8-GPS-1000/p/17005083) |
| Rainin 250ul Space Saver Tips | Anachem | £38 | £0.04 | [https://www.anachem.co.uk/](https://www.anachem.co.uk/Pipette-Tips/BioClean-Universal-Tips/SpaceSaver/Tips-1000-%C2%B5L-768-8-GPS-1000/p/17005083) |
| 10ul Graduated Tips | Starlabs | £29 | £0.03 | Personal contact via email with Starlabs |
| Pastettes | Alpha labs | £24 | £0.05 | [https://www.anachem.co.uk/](https://www.anachem.co.uk/Pipette-Tips/BioClean-Universal-Tips/SpaceSaver/Tips-1000-%C2%B5L-768-8-GPS-1000/p/17005083) |
| 1.5ml Screw Cap Tubes | Starlabs | £38 | £0.15 | Personal contact via email with Starlabs |
| 1.5ml Flip Cap Eppendorf Tubes | Starlabs | £20 | £0.04 | Personal contact via email with Starlabs |
| LoBind 1.5ml Eppendorf Tubes | Sigma Aldrich | £35 | £0.03 | [http://www.sigmaaldrich.com/catalog/product](http://www.sigmaaldrich.com/catalog/product/sigma/z606340?lang=en&region=GB&gclid=EAIaIQobChMIgOf19Pem1QIVDeMbCh1QrgbnEAAYASAAEgJG7_D_BwE) |
| BigDye V3.1 Sequencing Kit | Life Technologies (Applied Biosystems) | £7,410 | £7.41 | [https://www.thermofisher.com/order/](https://www.thermofisher.com/order/catalog/product/4336699?ICID=search-4336699) |
| Diluent, BigDye (v3.1) | Life Technologies (Applied Biosystems) | £904 | £0.06 | [https://www.thermofisher.com/order/](https://www.thermofisher.com/order/catalog/product/4336699?ICID=search-4336699) |
| FrameStar 96 PCR Plates Clear | 4TITUDE | £227 | £4.54 | Personal contact via email with 4TITUDE |
| Adhesive Heat Seal - Pierce Lids | 4TITUDE | £49 | £0.49 | Personal contact via email with 4TITUDE |
| Clean SEQ | Beckman Coulter | £2,466 | £0.49 | [http://uk.beckman.com/](http://uk.beckman.com/nucleic-acid-sample-prep/purification-clean-up/sanger-sequencing-dye-terminator-removal) |
| AMPure Beads (450ml) | Beckman Coulter | £4,043 | £0.45 | [http://uk.beckman.com/](http://uk.beckman.com/nucleic-acid-sample-prep/purification-clean-up/sanger-sequencing-dye-terminator-removal) |
| Agarose | Life Technologies (Invitrogen) | £267 | £0.89 | [https://www.thermofisher.com/order/](https://www.thermofisher.com/order/catalog/product/4336699?ICID=search-4336699) |
| TBE (10X) | Life Technologies (Invitrogen) | £106 | £0.21 | [https://www.thermofisher.com/order/](https://www.thermofisher.com/order/catalog/product/4336699?ICID=search-4336699) |
| 1Kb ladder | Life Technologies (Invitrogen) | £149 | £0.00 | [https://www.thermofisher.com/order/](https://www.thermofisher.com/order/catalog/product/4336699?ICID=search-4336699) |
| GoTaq Hot Start Green Master Mix, 100 | Promega | £536 | £1.40 | [https://www.promega.co.uk/products](https://www.promega.co.uk/products/pcr/endpoint-pcr/gotaq-hot-start-polymerase/?catNum=M5006) |
| Safeview Nucleic Acid Stain | NBS Biologicals | £179 | £0.18 | [http://www.nbsbio.co.uk](http://www.nbsbio.co.uk/nbs-sv5) |
| Qiagen EZ1 DNA Tissue Kit | Qiagen | £287 | £5.98 | [https://www.qiagen.com/gb/](https://www.qiagen.com/gb/shop/automated-solutions/sample-preparation/ez1-dna-tissue-kit/#orderinginformation) |
| SequalPrep Normalisation Kit | Life Technologies (Invitrogen) | £798 | £19.95 | <https://www.thermofisher.com/order> |
| Nextera XT Library Preparation Kit | Illumina | £2,521 | £26.26 | <https://www.illumina.com/products> |
| Nextera XT Index Kit v2 Set B | Illumina | £823 | £2.14 | <https://www.illumina.com/products> |
| MiSeq Reagent Kit v2 | Illumina | £974 | £0.06 | <https://www.illumina.com/products> |
| Qubit dsDNA HS Assay Kit | Life Technologies (Invitrogen) | £175 | £0.35 | [https://www.thermofisher.com/order/](https://www.thermofisher.com/order/catalog/product/4336699?ICID=search-4336699) |
| Qubit Assay Tubes | Life Technologies (Invitrogen) | £46.58 | £0.09 | [https://www.thermofisher.com/order/](https://www.thermofisher.com/order/catalog/product/4336699?ICID=search-4336699) |
| Primers | Sigma | £5 | £0.10 | Internal invoice |
| EtOH | Fluka | £111 | £2.23 | Internal invoice |
| Isopropanol | Fluka | £6 | £0.00 | Internal invoice |

|  |  |  |  |  |
| --- | --- | --- | --- | --- |

# Appendix 2- Results supplementary

## Pragmatic literature review

The pragmatic literature review used to inform the likelihood of positive results for each testing process identified 1119 manuscripts for title and abstract screening. Based on the selection criteria, 42 papers underwent full article review (Figure S2). In total 15 studies were identified that contained sufficient information that could generate test outcome probabilities. Of these, three were found to have low bias scores and therefore were used to inform the test probabilities within the four diagnostic strategies.

The most relevant primary source was the study by Goodfellow et al (1). This was due to the high quality of the study and use of all the 4 diagnostic tests. Based on the QUADAS2 score (<http://www.bristol.ac.uk/population-health-sciences/projects/quadas/quadas-2/>), two further studies were also used for sensitivity analysis (2,3).

### Table S3: Sensitivity analysis inputs from clinical studies identified through a pragmatic literature search.

Note: Only high quality studies as outlined in table S3 were used for sensitivity analysis – these include studies found to have a low risk of bias score with the use of the QUADAS-2 instrument (<http://www.bristol.ac.uk/population-health-sciences/projects/quadas/quadas-2/>)

| Outcome | Value | Source | Study Selection Criteria | Comments |
| --- | --- | --- | --- | --- |
| Proportion MSI-H | 22% | Hampel 2006 | None |  |
|  | 27% | Cook 2013 | <80 years |  |
|  | 30% | Goodfellow 2015 | Endometrioid pathology |  |
|  | 26% | Zauber 2010 | None |  |
|  | 44% | Yoon 2008 | None |  |
|  |  |  |  |  |
| Proportion MSI-H found to have LS | 6% | Goodfellow 2015 | Endometrioid pathology |  |
|  | 8% | Hampel 2006 | None |  |
|  | 13% | Yoon 2008 | None |  |
|  |  |  |  |  |
| Proportion MSI-H found to have normal MLH1 methylation | 44% | Hampel 2006 | None |  |
|  | 16% | Goodfellow | Endometrioid pathology |  |
|  | 44% | Zauber | None |  |
|  |  |  |  |  |
| Proportion normal MLH1 methylation found to have LS (MSI) | 44% | Goodfellow 2015 | Endometrioid pathology |  |
|  | 26% | Hampel 2006 | None |  |
|  | 62% | Egoavil 2013 | None |  |
|  |  |  |  |  |
| Proportion found to have MLH1 loss at IHC | 16% | Joehlin-Price 2014 | None |  |
|  | 27% | Goodfellow 2015 | Endometrioid pathology |  |
|  | 6% | Backes 2011 | None |  |
|  | 16% | Mills 2014 | None |  |
|  | 18% | Buchanan 2014 | None |  |
|  |  |  |  |  |
| Proportion of MLH1 loss at IHC found to be MLH1 methylation normal | 7% | Goodfellow 2015 | Endometrioid pathology |  |
|  | 2% | Mills 2014 | None |  |
|  | 16% | Buchanan 2014 | None |  |
|  | 5% | Watkins 2017 | None |  |
|  |  |  |  |  |
| Proportion normal MLH1 methylation found to have LS (IHC) | 11% | Goodfellow 2015 | Endometrioid pathology | Only 47 of 107 who should have had NGS had it |
|  | 16% | Mills 2014 | None | Only 21 of 52 had who should have NGS had it |
|  | 10% | Buchanan 2014 | None | Only 158 of 170 had who should have NGS had it |
|  | 10% | Watkins 2017 | None | Only 10 of 11 had who should have NGS had it |
|  |  |  |  |  |
| Proportion found to have either MSH2, MSH6 or PMS2 loss at IHC | 6% | Joehlin-Price 2014 | None |  |
|  | 8% | Goodfellow 2015 | Endometrioid pathology |  |
|  | 6% | Backes 2011 | None |  |
|  | 9% | Mills 2014 | None |  |
|  | 6% | Buchanan 2014 | None |  |
|  |  |  |  |  |
| Proportion of MSH2, MSH6 or PMS2 loss at IHC found to have LS | 8% | Backes 2011 | None | Only 8 of 47 had who should have NGS had it |
|  | 20% | Goodfellow 2015 | Endometrioid pathology | Only 47 of 107 who should have had NGS had it |
|  | 30% | Mills 2014 | None | Only 21 of 52 had who should have NGS had it |
|  | 44% | Buchanan 2014 | None | Only 158 of 170 had who should have NGS had it |
|  |  |  |  |  |
| Proportion of ECs found to have LS if direct NGS is used | 5% | Backes 2011 | None | Only 8 of 47 had who should have NGS had it therefore proportion estimated |
|  | 7% | Batte 2014 | None | Only 15 of 47 had who should have NGS had it therefore proportion estimated |
|  | 3% | Buchanan 2014 | None | Only 158 of 170 had who should have NGS had it therefore proportion estimated |
|  | 6% | Ferguson 2014 | None | All tested with NGS |
|  | 7% | Mas-Moya 2015 | None | Only 21 of 52 had who should have NGS had it therefore proportion estimated |
|  | 6% | Ring 2016 | None | All tested with NGS |
|  | 4% | Goodfellow 2015 | Endometrioid pathology | Only 47 of 107 who should have NGS had it therefore proportion estimated |
|  | 2% | Hampel 2006 | None | All tested with NGS |
|  | 2% | Watkins 2017 | None | Only 10 of 11 who should have NGS had it therefore proportion estimated |

### Figure S2: Prism chart of study identification and selection

**1119** Papers identified

**1119** Titles and abstracted screened by NR

**83** Full papers reviewed by independently by 2 reviewers

**15** Full papers included.

Wrong pathology- Not Lynch syndrome (n=87)

Wrong pathology- Not endometrial cancer (n=132)

Wrong Study type* (n=353)

Study of aetiology/pathogenesis (n=233)

Other (n=231)

Duplicate (n=5)

Known Lynch syndrome (n=5)

Review/Editorial (n=14)

Non-cancer study (n=5)

Poor quality/insufficient number of subjects (n=32)

Identification

Screening

Eligibility

Records in PubMed

(n=411)

Records in Embase

(n=386)

Records in Web of Science (n=301)

(n=782)

Records in CENTRAL (n=3)

(n=782)

**3** Papers found to have low bias

# Appendix 3 – Salary source data

### Table S4: BMA pay scales used to calculate medical doctors’ salary costs.

| Grade | Pay (£) |
| --- | --- |
| FY1 | 26,614 |
| FY2 | 30,805 |
| ST1 | 36,461 |
| ST2 | 36,461 |
| ST3 | 46,208 |
| ST4 | 46,208 |
| ST5 | 46,208 |
| ST6 | 46,208 |
| ST7 | 46,208 |
| ST8 | 46,208 |
| Consultant Yr 1 | 76,761 |
| Consultant Yr 2 | 79,165 |
| Consultant Yr 3 | 81,568 |
| Consultant Yr 4 | 83,972 |
| Consultant Yr 5-8 | 86,369 |
| Consultant Yr 9-13 | 92,078 |
| Consultant Yr 14-18 | 97,787 |
| Consultant Yr >19 | 103,490 |

NB: Consultant pay doesnt include clinical excellence awards

NB: Non-consultant pay does not include out of hours stipend

Source:

<https://www.bma.org.uk/advice/employment/pay/consultants-pay-england>

and

https://www.bma.org.uk/advice/employment/pay/juniors-pay-england

References:

1. Goodfellow PJ, Billingsley CC, Lankes HA, Ali S, Cohn DE, Broaddus RJ, et al. Combined Microsatellite Instability, MLH1 Methylation Analysis, and Immunohistochemistry for Lynch Syndrome Screening in Endometrial Cancers From GOG210: An NRG Oncology and Gynecologic Oncology Group Study. J Clin Oncol. American Society of Clinical Oncology; 2015 Dec 20;33(36):4301–8.

2. Hampel H, Frankel W, Panescu J, Lockman J, Sotamaa K, Fix D, et al. Screening for Lynch syndrome (hereditary nonpolyposis colorectal cancer) among endometrial cancer patients. Cancer Res. American Association for Cancer Research; 2006 Aug 1;66(15):7810–7.

3. Joehlin-Price AS, Perrino CM, Stephens J, Backes FJ, Goodfellow PJ, Cohn DE, et al. Mismatch repair protein expression in 1049 endometrial carcinomas, associations with body mass index, and other clinicopathologic variables. Gynecol Oncol. Elsevier; 2014 Apr;133(1):43–7.
